# A case of successful endoscopic ultrasound/endosonography-guided pancreaticogastrostomy using a novel plastic stent

**DOI:** 10.1055/a-2435-8199

**Published:** 2024-10-25

**Authors:** Daisuke Namima, Toshio Fujisawa, Yusuke Takasaki, Ko Tomishima, Shigeto Ishii, Hideki Kobara, Hiroyuki Isayama

**Affiliations:** 1 Department of Gastroenterology, Juntendo University, Graduate School of Medicine, Tokyo, Japan; 2 12850 Department of Gastroenterology and Neurology, Kagawa University, Kagawa, Japan

Endoscopic ultrasound-guided pancreaticogastrostomy (EUS-PGS) for calcified chronic pancreatitis is challenging, particularly when it involves insertion of a plastic stent. Therefore, there is a need for an easily insertable dedicated plastic stent 1 2 3 4 5 . We have developed an integrated plastic stent with an inner catheter and a pull-back mechanism, and to sharpen the plastic stent tip, the inner catheter is inserted only 10 mm beyond the plastic stent tip. The insertion system is novel: the plastic stent is pushed by both the pushing catheter (lower end of the stent) and the inner catheter (near the tip of the stent) ( Fig. 1 ). Together with the sharper tip, these features facilitate pushing of the plastic stent. Additionally, the flaps of the new plastic stent are attached to its body to prevent stent migration ( Fig. 2 ).

**Fig. 1:**
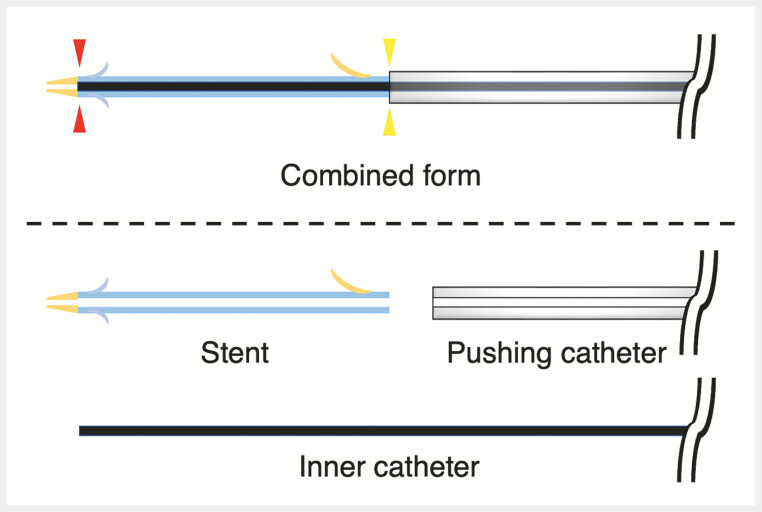
A newly developed integrated plastic stent. The stent can be pushed from two points: the outer catheter (yellow arrowhead) and the inner catheter (red arrowhead).

**Fig. 2:**
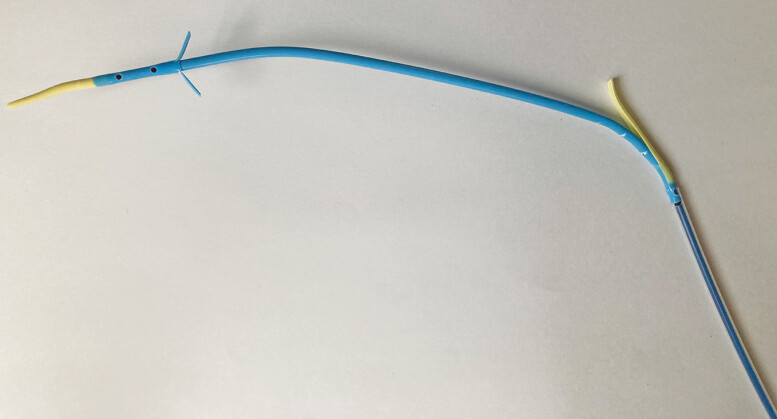
The photograph shows the new plastic stent; the tip is tapered and equipped with large flaps.

We performed EUS-PGS in a 68-year-old man with calcified chronic pancreatitis ( Fig. 3 ) in whom an attempt to pass the stricture by the transpapillary route had failed at another hospital. The main pancreatic duct was punctured using a 19-gauge needle, and a 0.025-inch guidewire was inserted past the pancreatic-head stricture. Subsequently, the puncture tract and the stricture were dilated using a 7-Fr drill dilator (Tornus; Olympus Medical Systems, Tokyo, Japan) and a 4-mm balloon catheter (Ren; Kaneka Medical Devices, Tokyo, Japan). While inserting a 7-Fr double-pigtail plastic stent (Piglet, Olympus Medical Systems), the inner catheter passed the stricture, but the stent did not, even after additional balloon dilation. We attempted to pass the stricture using the prototype of the new plastic stent; the attempt was successful. Finally, we placed the tip of the new plastic stent in the duodenum ( Video 1 ). EUS-PGS for calcified chronic pancreatitis was hampered by hard parenchyma and the tightness of the stricture. In this case, the new, easy-to-push, anchored plastic stent enabled safe and effective EUS-PGS. More clinical data on the effectiveness of the new plastic stent are needed.

**Fig. 3:**
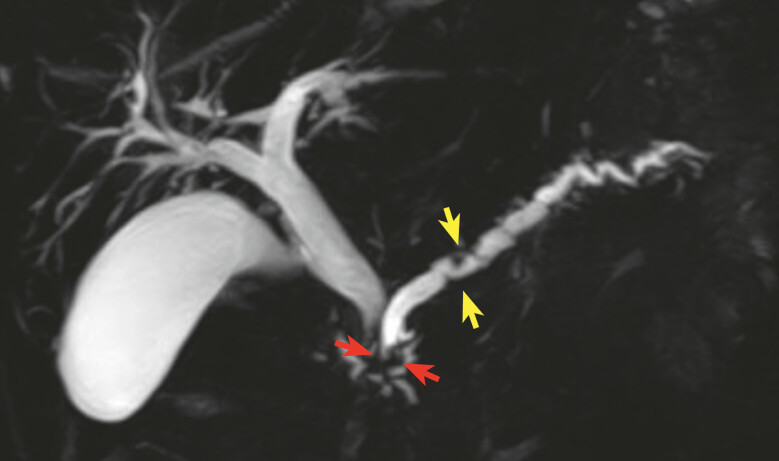
Magnetic resonance cholangiopancreatography shows strictures and dilatations of the main pancreatic duct; the stricture near the papilla (red arrow) is more severe than the stricture in the body (yellow arrow).

A case of successful endoscopic ultrasound-guided pancreaticogastrostomy using a novel plastic stent.Video 1

Endoscopy_UCTN_Code_TTT_1AS_2AD

## Notes

Endoscopy E-Videos https://eref.thieme.de/e-videos
*E-Videos* is an open access online section of the journal *Endoscopy* , reporting on interesting cases and new techniques in gastroenterological endoscopy. All papers include a high-quality video and are published with a Creative Commons CC-BY license. Endoscopy E-Videos qualify for HINARI discounts and waivers and eligibility is automatically checked during the submission process. We grant 100% waivers to articles whose corresponding authors are based in Group A countries and 50% waivers to those who are based in Group B countries as classified by Research4Life (see: https://www.research4life.org/access/eligibility/ ). This section has its own submission website at https://mc.manuscriptcentral.com/e-videos .
